# Domestication-driven changes in plant traits associated with changes in the assembly of the rhizosphere microbiota in tetraploid wheat

**DOI:** 10.1038/s41598-020-69175-9

**Published:** 2020-07-22

**Authors:** Aymé Spor, Agathe Roucou, Arnaud Mounier, David Bru, Marie-Christine Breuil, Florian Fort, Denis Vile, Pierre Roumet, Laurent Philippot, Cyrille Violle

**Affiliations:** 1 Université Bourgogne Franche-Comté, INRAE, AgroSup Dijon, Agroécologie, 21000 Dijon, France; 2 CEFE, Univ Montpellier, CNRS, EPHE, IRD, Université Paul Valéry, Montpellier, France; 3 LEPSE, INRAE, Institut Agro, Univ Montpellier, Montpellier, France; 4 Agroécologie, AgroSup Dijon, CNRS, INRAE, Univ. Bourgogne, Univ. Bourgogne Franche-Comté, 21000 Dijon, France; 5 CEFE, Univ Montpellier, CNRS, EPHE, Institut Agro, IRD, Université Paul Valéry, Montpellier, France; 6 AGAP, Univ Montpellier, CIRAD, INRAE, Institut Agro, Montpellier, France

**Keywords:** Microbial communities, Ecology

## Abstract

Despite the large morphological and physiological changes that plants have undergone through domestication, little is known about their impact on their microbiome. Here we characterized rhizospheric bacterial and fungal communities as well as the abundance of N-cycling microbial guilds across thirty-nine accessions of tetraploid wheat, *Triticum turgidum*, from four domestication groups ranging from the wild subspecies to the semi dwarf elite cultivars. We identified several microbial phylotypes displaying significant variation in their relative abundance depending on the wheat domestication group with a stronger impact of domestication on fungi. The relative abundance of potential fungal plant pathogens belonging to the Sordariomycetes class decreased in domesticated compared to wild emmer while the opposite was found for members of the Glomeromycetes, which are obligate plant symbionts**.** The depletion of nitrifiers and of arbuscular mycorrhizal fungi in elite wheat cultivars compared to primitive domesticated forms suggests that the Green Revolution has decreased the coupling between plant and rhizosphere microbes that are potentially important for plant nutrient availability. Both plant diameter and fine root percentage exhibited the highest number of associations with microbial taxa, highlighting their putative role in shaping the rhizosphere microbiota during domestication. Aside from domestication, significant variation of bacterial and fungal community composition was found among accessions within each domestication group. In particular, the relative abundances of Ophiostomataceae and of Rhizobiales were strongly dependent on the host accession, with heritability estimates of ~ 27% and ~ 25%, indicating that there might be room for genetic improvement via introgression of ancestral plant rhizosphere-beneficial microbe associations.

## Introduction

Crop domestication events led to spectacular modifications of plant traits increasing their suitability to human requirements. Cereals and especially wheat have been domesticated in the Middle East^1^. Domestication process led to dramatic phenotypic changes in cultivated species in relation to cultivation conditions and human needs. The Domestication syndrome^2^ refers to the whole set of phenotypic changes occurred during this process, including: loss of dormancy, increasing seed size, modifying seed dispersal mode and apical dominance as well photoperiod sensitivity.

Domestication of tetraploid wheat (wild emmer, *Triticum turgidum* ssp. *Dicoccoides *(*Körn.*),* Thell*.) firstly led to a non-brittle hulled subspecies (cultivated emmer, *T. t.* ssp. *Dicoccum Schrank*) which was one of the first cereals domesticated^1^*.* Then, human selection of mutations at multiple loci, notably at *Tg*—tenacious glume—and *Q* loci^3–5^, led to the evolution of traits such as soft glumes, non-hulled grains improving threshing efficiency^6^ and facilitating its cultivation in various environments. This phenotypic evolution together with hybridization between different forms^7^ led to free-threshing subspecies (*T. t.* ssp. *polonicum*,* T. t.* ssp. *carthlicum*,* T. t.* ssp. *turgidum*,* T. t.* ssp. *Durum *(*Desf*.)* MacKey*) that were widely spread out with the early agricultural movements. Nowadays, elite durum varieties and landraces from *T. t. durum* grown in different environments are of major importance for grain production in the Mediterranean basin^8^. Although domestication is a continuous process including modern revolution and modern plant breeding, Gioia et al*.*^9^ suggested to distinguish a primary domestication period (from wild emmer to cultivated emmer) from a secondary one’s (from cultivated emmer to the free-threshing subspecies including durum wheat). Molecular markers-based studies showed that *durum* wheat history was associated with a significant decrease of the level of genetic diversity, from wild ancestors to the most recent modern varieties^10,11^. It has also been speculated that domestication-driven phenotypic changes have profoundly altered above and belowground biotic interactions, notably because they induce changes in the associated microbiota habitat^12^. This is also strengthened by the fact that domesticated crops during the Green Revolution were selected for agroecosystems characterized by an extensive use of pesticides and fertilizers, and are therefore less prone to rely on their associated microbiota for their growth.

Rhizosphere microbes are important for plants because they provide benefits to their host. Both bacteria and fungi, in particular symbiotic rhizobia and arbuscular mycorrhizal fungi (AMF), are known to mediate nutrient acquisition such as nitrogen, iron or phosphorus (reviewed in Ref.^13^), enhance plant tolerance to environmental stresses^14^ and protect against pathogens by acting as the first line of defence against soil-borne pathogens^15^. In turn, roots secrete large amounts of rhizodeposits that can be utilized as carbon sources by microbes for growth and development, but also secondary metabolites that can be important for establishing symbiosis with microbes or acting against plant pathogens invasion. Recent literature describing the effect of plant genotypes on their associated rhizosphere microbiota revealed in some species that an heritable intraspecific variation driven by the host’s genetics exists^16–20^. However, little is known about the extent of the impact of plant domestication on the rhizosphere microbiota. When analysing a handful of genotypes of wild and domesticated barley or *Phaseolus vulgaris*, Bulgarelli et al*.*^21^ and Perez-Jaramillo et al*.*^22^ reported small but significant differences in rhizosphere bacterial composition associated with differences in host genotypes. On the opposite, in a more comprehensive study based on 33 sunflower genotypes that varied in their extent of domestication, Leff et al*.* demonstrated that domestication affected only rhizosphere and seed associated fungal communities and neither root nor rhizosphere bacterial communities^23^.

Here, we took advantage of a unique collection of genotypes belonging to three main sub-species representative of tetraploid wheat domestication. These genotypes have been characterized for a variety of above and belowground morphological traits, and have been shown to display contrasted ecological strategies depending on domestication^24^. We characterized the rhizosphere microbiota of 39 selected accessions, homogeneously divided in four representative accession subsets corresponding to four main steps of the evolutionary history of *Triticum turgidum *i.e. wild, first domesticated, free threshing landraces and post Green Revolution elite lines respectively. We hypothesized that wheat domestication, by modifying key crop traits, might have weakened the association between wheat and its rhizosphere microbiota, and therefore led to significant changes in rhizosphere bacterial and fungal communities.

## Materials and methods

### Plant material

Forty accessions of tetraploid wheat were selected from the four domestication series groups represented by the three subspecies *T. turgidum *ssp*. dicoccoides *(*Körn*)* Thell*, wild emmer), *T. turgidum *ssp.* dicoccum*, (cultivated emmer), and *T. turgidum *ssp*. Durum *(*Desf*)* MacKey*. This last sub-species was split in two subgroups to distinguish pre *versus* post Green Revolution durum wheat accessions; the first subgroup is issued from landraces and the second one related to semi dwarf elite accessions registered in Europe after the Green Revolution (from 1970s to 1990s) (Supplementary Table S1).

For each group, ten accessions have been sampled to maximize the genetic diversity of each group based on 21 non-linked microsatellite markers mapped on 14 *durum* chromosomes^25^. The accessions came from seed collections maintained in different stock centers (see Supplementary Table S1). For each accession, seeds came from successive self-fertilizations realized in common gardens to limit residual heterozygosity and to ensure that the material is genetically fixed. Seeds were provided by INRAE UMR AGAP in Montpellier.

### Experimental design

We set up the experiment at the INRAE-SupAgro campus (France, 43° 37′ 02″ N, 3° 51′ 18″ E) for a duration of 90 days in outdoor conditions (minimal temperature = 2 °C and maximal temperature = 19 °C). As described in Ref.^24^, plants were grown in 9 L pots (17.5 cm diameter and 38.5 cm depth) filled with 2 mm sieved topsoil taken from a natural grassland (France, 44° 26′ 25″ N, 3° 55′ 58″ E; 0.994 g N kg^−1^ soil, 4.17 g K kg^−1^ soil and 0.166 g P kg^−1^ soil). To synchronize germination, we put seeds into Petri dishes for 3 days at 4 °C, and 4 days at ambient temperature. After germination, two seedlings were transplanted (at 2 cm depth) in each pot and thinned to one plant per pot at the first leaf stage. We performed a randomized complete block design using five blocks, where each accession was represented by one plant in each block. To avoid water stress, plants were watered once or three times per week with 150 mL of tap water, according to climatic conditions. One accession from *T. turgidum* ssp. *dicoccoides* did not grow up during the experiment. We therefore removed it from the following analyses (Supplementary Table S1).

At vegetative stage, we determined traits on each plant. Aboveground traits were measured at the leaf level: leaf nitrogen content (LNC, %) and leaf longevity (LL, °Cd), and at the plant level: plant diameter (PD, cm) and shoot dry mass (SDM, g). At harvest, belowground parts were extracted from soil; architectural trait, crow root angle (RA, °), and morphological traits, percent of fine roots (%), mean root diameter (RD, mm) and root dry mass (RDM, g) were determined after rhizosphere soil samples collection (see below) according to the detailed acquisition protocol reported in Ref.^24^.

### Rhizosphere soil samples collection

Plants were manually taken off the pots and shaken vigorously to remove loose soil. Roots were then cut and washed twice with 100 mL KCL 1 N in 500 mL sterile centrifuge pots shaken at 350 rpm. The resulting slurry was then centrifuged at 4,000*g*. The supernatant was removed and used for quantification of nitrogen pools. The same procedure was used for bulk soil samples. Bulk soil samples are samples coming from pots without any plants (five replicates) and from planted pots from eight genotypes (two per subgroup) replicated five times. We therefore have 195 rhizosphere samples and 45 bulk soil samples. The soil pellet was used for subsequent DNA extractions.

### Amplicon generation and MiSeq sequencing

Amplicons were generated in two steps according to Ref.^26^. In the first step, the bacterial 16S rRNA gene V3–V4 hypervariable region was amplified by polymerase chain reaction (PCR) using the fusion primers U341F (5′-CCTACGGGRSGCAGCAG-3′) and 805R (5′-GACTACCAGGGTATCTAAT-3′)^27^, with overhang adapters (forward: TCGTCGGCAGCGTCAGATGTGTATAAGAGACAG, adapter: GTCTCGTGGGCTCGGAGATGTGTATAAGAGACAG) to allow the subsequent addition of multiplexing index-sequences. PCR was carried out in duplicate 15 µL reactions containing 7.5 µL Phusion High-Fidelity PCR Master Mix (THERMO FISHER SCIENTIFIC), 0.25 µM of each primer, 250 ng T4 gp32 (MPBIO) and 1 ng template DNA. Thermal cycling conditions were 98 °C for 3 min followed by 25 cycles of 98 °C for 30 s, 55 °C for 30 s and 72 °C for 30 s, with a final extension at 72 °C for 10 min. Duplicate first step PCR products were pooled then used as template for the second step PCR. In the second step, PCR amplification added multiplexing index-sequences to the overhang adapters using a unique multiplex primer pair combination for each sample. The reaction was carried out in duplicate 30 µL volumes containing 15 µL Phusion High-Fidelity PCR Master Mix (THERMO FISHER SCIENTIFIC), 1 µM of one forward and one reverse multiplex primer and 6 µL of first step PCR product. Thermal cycling conditions were 98 °C for 3 min followed by 8 cycles of 98 °C for 30 s, 55 °C for 30 s and 72 °C for 30 s, with a final extension at 72 °C for 10 min. Duplicate second step PCR products were pooled and then visualized in 2% agarose gel to verify amplification and their sizes. The amplicons were cleaned-up and pooled using SequalPrep Normalization plate kit 96-well (INVITROGEN). Sequencing was performed on MiSeq (ILLUMINA, 2 × 250 bp) using the MiSeq reagent kit v2 (500cycles). Demultiplexing and trimming of ILLUMINA adaptors and barcodes was done with ILLUMINA MiSeq Reporter software (version 2.5.1.3). Fungal ITS rRNA region was amplified similarly, using the primers ITS3F (5′-GCATCGATGAAGAACGCAGC-3′) and ITS4R (5′-TCCTCSSCTTATTGATATGC-3′), modified from Ref.^29^, with 30 cycles for the first step PCR and 10 cycles for the second step PCR.

### Quantification of total and N-cycle related microbial communities

DNA was extracted from 250 mg dry-weight soil for all 240 samples using the DNeasy PowerSoil-htp 96 well DNA isolation kit (QIAGEN, France).Total bacterial communities were quantified using 16S rDNA and ITS primers-based qPCR assays as described in Refs.^28,29^. Quantification of the bacterial and archaeal ammonia-oxidizers (AOB and AOA respectively) was performed according to Refs.^30^^,^^31^ whereas quantification of denitrifiers was performed according to Refs.^32,33^^,^^34^. For this purpose, the genes encoding catalytic enzymes involved in nitrogen fixation (*nifH*), ammonia-oxidation (bacterial and archaeal *amoA*), nitrite reduction (*nirK* and *nirS*) and nitrous oxide reduction (*nosZ*I and *nosZ*II) were used as molecular markers. Reactions were carried out in a ViiA7 (LIFE TECHNOLOGIES, USA). Quantification was based on the increasing fluorescence intensity of the SYBR Green dye during amplification. The real-time PCR assays were carried out in a 15 µL reaction volume containing SYBR green PCR Master Mix (Absolute Blue QPCR SYBR Green Low Rox Mix, THERMO FISHER SCIENTIFIC, France), 1 µM of each primer, 250 ng of T4 gene 32 (QBIOGENE, France) and 0.5 ng of DNA as previously described^35^. Three independent replicates were considered for real time PCR assays. Standard curves were done using serial dilutions of linearized plasmids containing appropriate cloned targeted genes from bacterial strains or environmental clones. PCR efficiency for the different assays ranged from 70 to 99%. No template controls gave null values. The presence of PCR inhibitors in DNA extracted from soil and rhizosphere samples was estimated by mixing a known amount of standard DNA with soil DNA extract before qPCR. We did not detect inhibition in any case.

### Bioinformatic analysis of the 16S rRNA and ITS amplicons

Sequence data were analysed using an in-house developed Python notebook piping together different bioinformatics tools (available upon request). Briefly, for both 16S and ITS data, sequences were assembled using PEAR^36^ with default settings. Further quality checks were conducted using the QIIME pipeline^37^ and short sequences were removed (< 335 bp for 16S and < 310 bp for ITS). Reference based and de novo chimera detection, as well as clustering in OTUs were performed using VSEARCH^38^ and the adequate reference databases (Greengenes’ representative set of sequences for 16S and UNITE’s ITS2 reference dynamic dataset for ITS). The identity thresholds were set at 97% for 16S and ITS data. For 16S rRNA data, representative sequences for each OTU were aligned using PYNAST^39^ and a 16S rRNA phylogenetic tree was constructed using FASTTREE^40^ Taxonomy was assigned using UCLUST^41^ and the latest released Greengenes database (v.05/2013^42^) for 16S rRNA. For ITS, the taxonomy assignment was performed using BLAST^43^ and the UNITE reference database (v.7-08/2016^44^). Raw sequences were deposited at the NCBI under the accession number SUB6424930. The process of raw sequence submission was performed using the *make.sra* command of MOTHUR software.

Bacterial and fungal α-diversity metrics describing the structure of microbial communities included indices pertaining to richness (observed species, chao1), evenness (Simpson reciprocal), both (Shannon), and relatedness (Faith’s Phylogenetic Diversity PD; (Faith, 1992) for bacterial community data only) and were calculated based on rarefied OTU tables (12,000 sequences and 20,000 sequences for 16S rRNA and ITS respectively). Bray–Curtis dissimilarity matrices were also computed to detect global variations in the composition of microbial communities for 16S rRNA and ITS. Bacterial co-occurrence networks were constructed for bulk soil, and the four subspecies separately in order to detect associations between bacteria that might have been lost or might have emerged during the course of domestication. Co-occurence or exclusion between two OTUs was considered as relevant when Spearman correlation coefficient was significant and > 0.6 or < − 0.6. Because Spearman correlations are known to be sensitive to zeros, only the most abundant OTUs were kept for this analysis. Also, several other metrics could have been used to detect correlations between OTUs, however, the aim of the present analysis being to compare networks across the four domestication groups, we believe that the choice of the metrics is less important than if it was to compare it to existing knowledge.

### Statistical analyses

The analyses of the different response variables (diversity indices, QPCRs abundance data, OTUs relative abundance data) was done using general mixed models. Response variables were log10-transformed prior to analysis. To detect differences in response variables related to the environmental compartment (rhizosphere versus bulk soil), the following model was used:

$${Y}_{ijk}=\mu +{Block}_{i}+{compartment}_{j}+{\varepsilon }_{ijk}$$

where Y_ijk_ is the response variable, Block is the random Block effect (i = 1, …, 5), compartment is a fixed compartment effect (j = 1, 2) and *є*_*ijk*_ is the residuals.

To detect differences in response variables related to domestication, or to the host genotypic variation, the following model was used on rhizosphere data:

$${Y}_{ijkl}=\mu +{Block}_{i}+{subspecies}_{j}+Genotype{\left(subspecies\right)}_{jk}+{\varepsilon }_{ijkl}$$

where Y_ijkl_ is the response variable, Block is the random Block effect (i = 1, …, 5), subspecies is a fixed subspecies effect (j = 1, …, 4) depicting the domestication effect, Genotype(subspecies) is the random genotypic effect nested within each subspecies (k = 1, …, 10) and *є*_*ijkl*_ is the residuals. For all studied response variables, normality and homoscedasticity of the residuals distribution was visually inspected. Note that we opted for a custom mixed model analysis to take into account the relative complexity of our experimental design that cannot be handled by classical analysis packages such as edgeR or DESeq2. A False Discovery Rate procedure was used to decrease substantially the probability to detect false positive. Broad sense heritability estimates were calculated as the part of variance explained by the Genotype (subspecies) effect divided by the total variance.

Constrained Principal Coordinate Analyses were performed to retrieve the amount of variation of total bacterial and fungal communities explained by the different explaining variables (compartment, subspecies, Genotype) when conditioning for Block using the vegan *capscale* function. Finally, Mantel tests were performed to detect correlations between microbial communities data (Bray–Curtis distance matrices) and plant phenotypic data (Euclidean distance matrix).

Integration and combined visualization of microbial and plant phenotypic data sets were realized using the R package mixOmics^45^ using DIABLO (Data Integration Analysis for Biomarker discovery using a Latent component method for Omics studies) in order to identify correlated key variables in both datasets^46^ explaining differences between subspecies.

## Results and discussion

### Wheat rhizosphere shapes the soil microbiota

Like many other plant species, wheat affected the microbiome assembly in the rhizosphere. Quantification of the abundance of the bacterial communities showed significant differences between the wheat rhizosphere and the soil compartments (p < 0.05). We also observed a significant effect of the compartment (rhizosphere soil vs bulk soil) on the number of bacterial species (observed OTUs, p < 0.01) as well as on the Simpson reciprocal index for both bacterial and fungal communities (p < 0.01). In addition to the changes in *α*-diversity, 

clear differences between

 compartments in microbial community composition were also found (p < 0.05, Supplementary Fig S1a,b). At the class level, several bacterial taxa exhibited contrasted relative abundances between the soil and the rhizosphere (Supplementary Fig S1c,d). Rhizosphere samples were enriched in members of the *α*- and γ-proteobacteria, flavobacteriia, cytophagia and actinobacteria classes and depleted in members of the gemmatimonadetes, acidobacteria phylum and thermoleophilia class compared to bulk soil. Among fungi, members of the Eurtiomycetes class were more abundant in the rhizosphere while members of the Leotiomycetes, Pezizomycetes, Tremellomycetes and Mortierellomycotina were over-represented in the soil. These results confirm previous findings that the rhizosphere is a selective environment which favours growth of potentially adapted or fast growing microorganisms^17,47^.

### Domestication affected specific bacterial and fungal taxa in the rhizosphere

**Figure 1:**
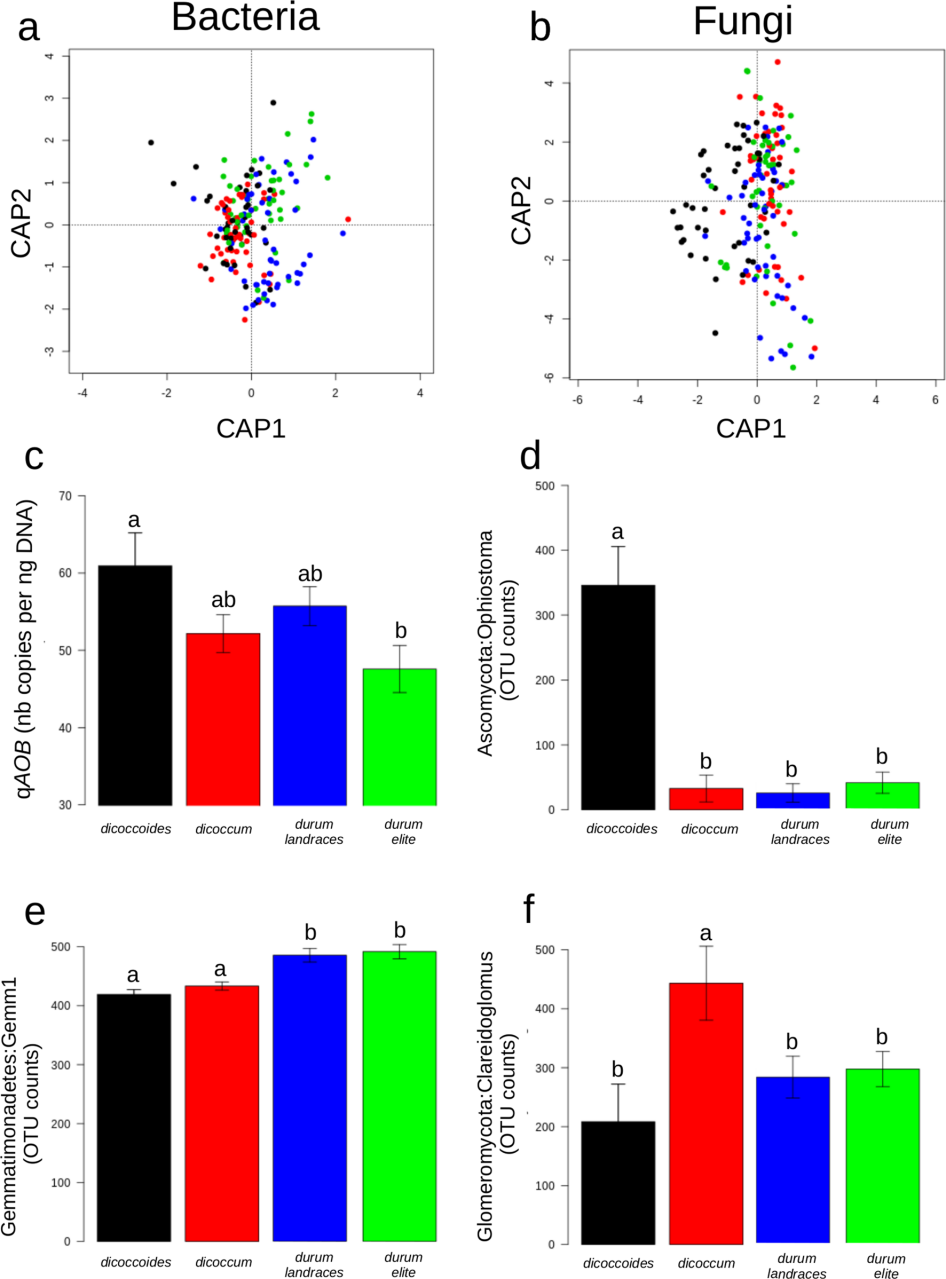
Impact of domestication on bacterial and fungal communities composition. Constrained analyses of principal coordinates of bacterial (**a**) and fungal (**b**) communities. Black, red, green and blue dots correspond to *T. turgidum *spp*. dicoccoides*, *T. turgidum *spp.* dicoccum*, *T. turgidum *spp.* durum* landraces and *T. turgidum *spp.* durum* elite cultivars, respectively. (**c**) Mean gene copy numbers (and standard deviations) of ammonia-oxidizing bacteria across the four domestication groups expressed in number of copies per g of DNA. Same colors as above. (**d**–**f**) Distribution of counts assigned to Ascomycota:Ophiostoma, Gemmatimonadetes:Gemm1 and Glomeromycota:Claroideoglomus, respectively, across the four domestication groups. Mean and standard errors are represented. Same colors as above.

We detected no changes in bacterial and fungal *α*-diversity indices related to domestication contrary to what is classically observed in the literature, probably related to larger number of accessions within each domestication group. A small but significant proportion of the total variance in the structure of the bacterial and fungal communities was attributable to domestication (4.1%, p = 0.001 and 3.4%, p = 0.002 respectively for bacteria and fungi; Fig. 1a,b). The sizes of the total bacterial and fungal communities were equivalent across the four different wheat groups. AOB was the only N-cycle guild whose abundance was significantly impacted by domestication with a decrease along the domestication gradient (wild emmer > cultivated emmer > durum wheat elite genotypes, Fig. 1c), with the durum landraces genotypes being in-between wild emmer and elite genotypes. When analysing the relative abundance of members of the fungal community, we found two clear contrasted patterns across the domestication series. The first pattern, consisting in a strong decrease in relative abundance between wild emmer and the three domestication groups, was detected for four members of the Sordariomycetes class: the *Togniniella*, the *Chaetosphaeria*, the *Ophiostoma* and the *Raffaela* genera, as well as for one member of the Chytridiomycetes class: the *Clydaea* genus (Fig. 1d, Supplementary Fig. S2a). Interestingly, several members of these genera have been found to be pathogens associated to plant diseases (dutch elm disease for *Ophiostoma*^48^, oak and laurel wilt disease for *Raffaela*^49,50^, or grapevine trunk disease for *Togniniella*^51^). On the opposite, most of the members of the Glomeromycetes class showed the second pattern with increased abundance in cultivated emmer compared to wild emmer. This was the cases of the *Clareidoglomus*, the *Archaeospora* and an unclassified genera belonging to the Glomerales order (Fig. 1f, Supplementary Fig. S2b). All members of this order are obligate plant symbionts, known as arbuscular mycorrhizal fungi (AMF), which are colonizing host plant root for reciprocal nutrient exchange^52^. Our findings suggest that the association between AMF and cultivated emmer increased during the first domestication events, at the expense of potentially pathogenic fungi. Accordingly, previous studies suggested that AMF can provide systemic protection against a wide range of pathogens in several plants through “mycorrhiza-induced resistance”^14,53^. Interestingly, another member of the Glomeromycetes class, *Paraglomus,* displayed a strong abundance decrease between wild emmer compared to the three domestication groups (Supplementary Fig. S2a), which supports previous findings suggesting that *Paraglomus* spp*.* have distinct ecological characteristics compared to members of the glomerales order^54^.

About 20 bacterial phylotypes displayed significant variation in their relative abundance across the four tetraploid wheat domestication groups. Among those, two out of the three most abundant ones were members of the Flavobacteriaceae and Comamonadaceae families whose abundance were higher in wild emmer, cultivated emmer and elite lines than in landraces accessions. The third phylotype, the most abundant one, was classified as similar to the Gemm 1 clone (Fig. 1e) of the Gemmatimonadetes phylum. The relative abundance of this phylotype displayed a significantly increased abundance in modern durum cultivars compared to durum landraces. In contrast to Gemmatimonadetes, Comamonadaceae and Flavobacteria are often described as bacterial phylotypes exhibiting a significant rhizosphere effect^55–57^.

### Assembly co-ocurrence networks across wheat domestication groups of the rhizosphere bacterial community

**Figure 2:**
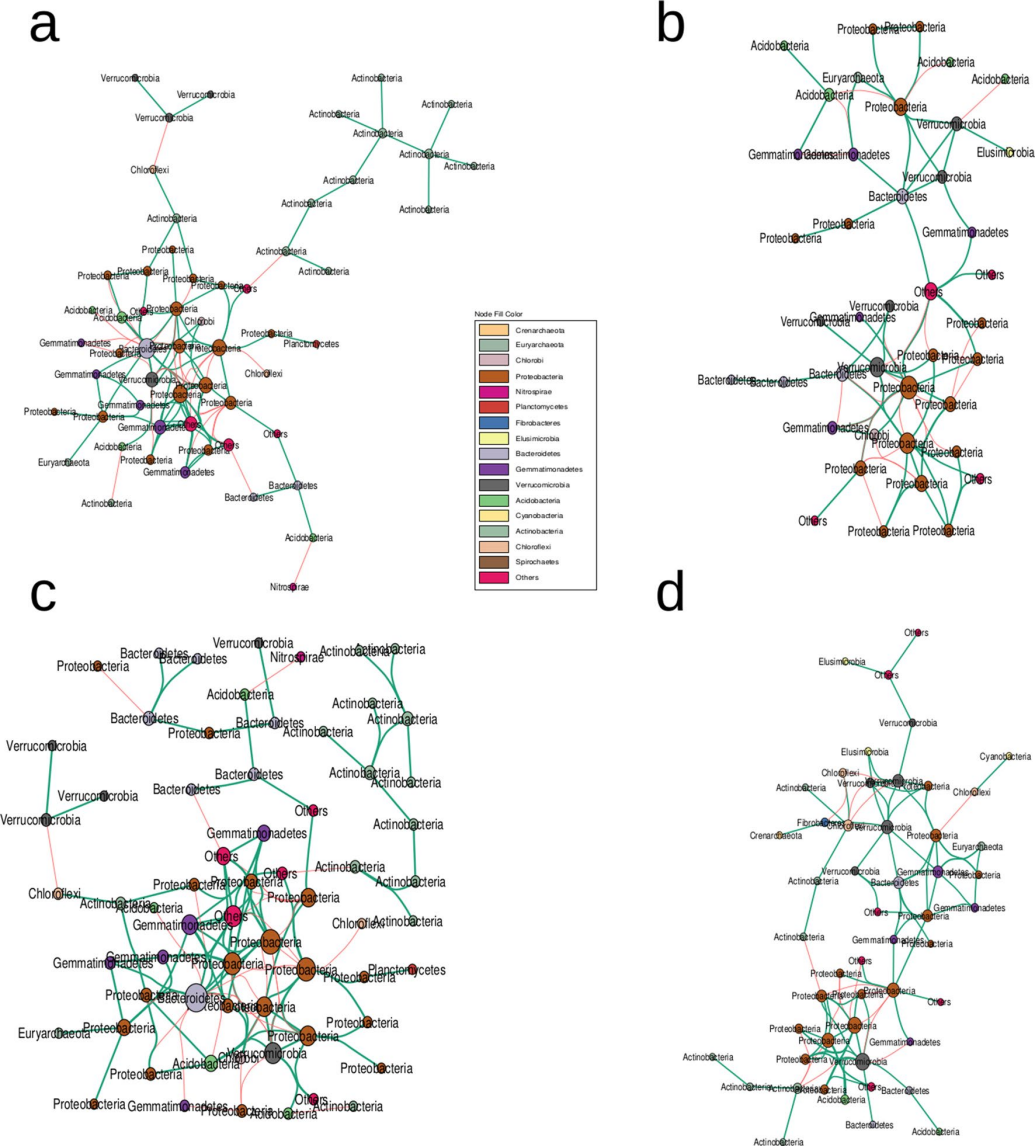
Bacterial cooccurence networks across the four domescation groups. (**a**–**d**) Correspond to *T. turgidum *spp.* dicoccoides*, *T. turgidum *spp.* dicoccum*, *T. turgidum *spp.* durum* landraces and *T. turgidum *spp.* durum* elite cultivars rhizosphere bacterial cooccurence networks, respectively. Edges indicate positive (green) or negative correlations (red) as defined by Spearmann’s correlation coefficients ρ > 0.6 or ρ < − 0.6, respectively. Node colors differ according to OTUs phylum assignment.

While no significant associations between fungal OTUs have been observed, a comparison of the co-occurence networks inferred for bacterial community from the four domestication groups and bulk soil revealed that a large part of those network members are shared between the four groups (Fig. 2 and Supplementary Fig. S3). Specifically, 24 bacterial nodes were present across all four subspecies networks amongst which 8 were unique to the rhizosphere (i.e. absent in the bulk soil network, Supplementary Fig. S3). Interestingly, most of the bacterial phylotypes whose abundances have been shown to be dependent on the host genotype are present in those co-occurence networks. While the majority of bacterial nodes composing the bulk soil network are bulk soil-specific, very few OTUs are domestication groups-network specific (none for *T. turgidum spp. dicoccoides*, 7 out of 41 for *T. turgidum* spp. *dicoccum*, 7 out of 51 for durum landraces and 1 out of 64 for durum elite accessions) which tends to indicate that domestication did not drastically change bacterial association patterns in the rhizosphere (Supplementary Fig. S3b).

### Aside from domestication, genotypic variability is a strong driver of rhizosphere bacterial and fungal community composition

**Figure 3:**
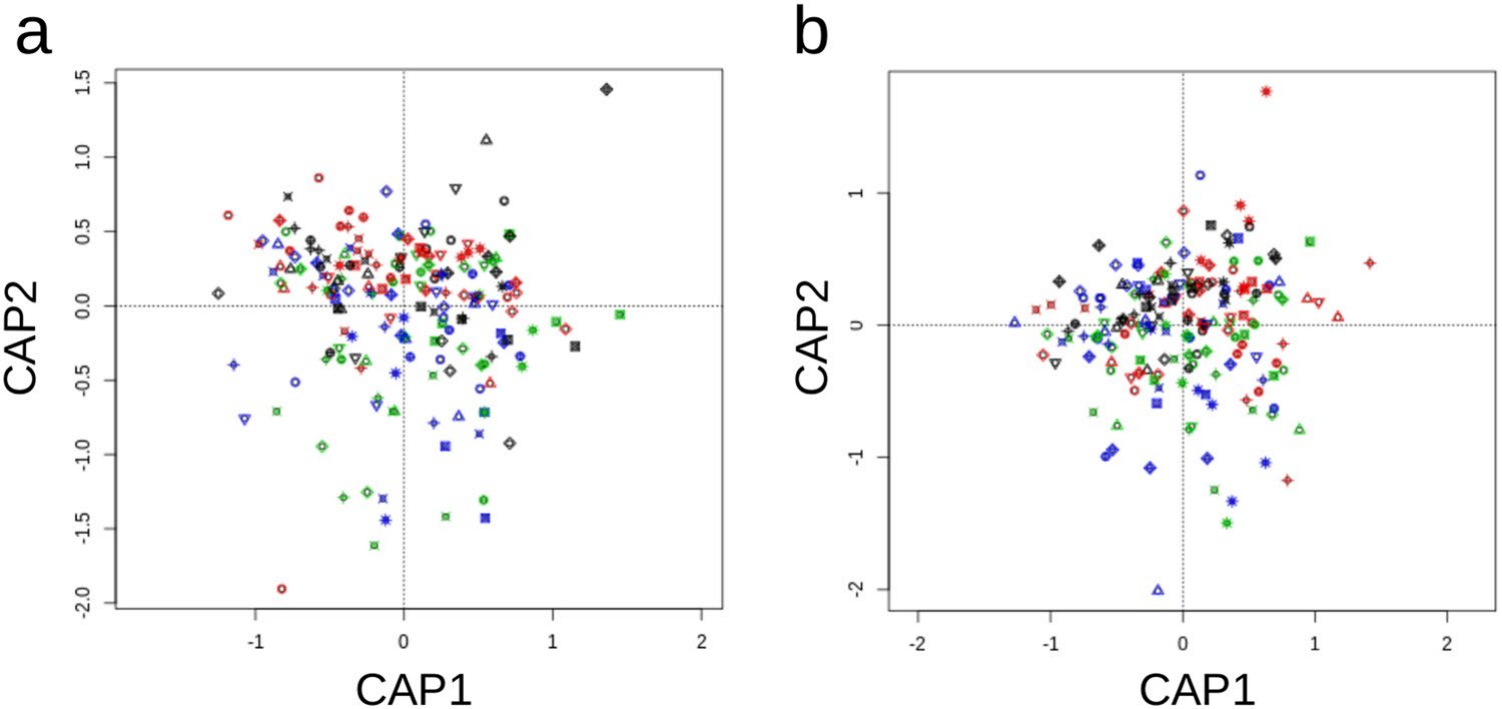
Impact of within-domestication group genotypic variation on bacterial and fungal communities composition. Constrained analyses of principal coordinates of bacterial (**a**) and fungal (**b**) communities. Black, red, green and blue dots correspond to *T. turgidum *spp.* dicoccoides*, *T. turgidum *spp.* dicoccum*, *T. turgidum *spp.* durum* landraces and *T. turgidum *spp.* durum* elite cultivars, respectively. Genotypes are encoded with different symbols within each domestication group.

A significant part of the observed variations in bacterial and fungal *α*-diversity indices was attributable to genotype variation within each subspecies. The broad sense heritability of the bacterial observed species and Simpson reciprocal indices were respectively 16.8% and 17.6% (p = 0.016 and p = 0.011). In contrast, estimates were not significant for the fungal observed species and Simpson reciprocal index. However, the composition of both bacterial and fungal communities in the rhizosphere was significantly under the influence of the host genotype. About 30% of the total variation in rhizosphere microbial communities is attributable to genotype variation (CAP analyses of Bray–Curtis distance matrices, 28.5%, p = 0.001 and 30.3%, p = 0.001 respectively for bacteria and fungi; Fig. 3a,b). At the global scale, within-domestication group variation in rhizosphere microbial communities is therefore stronger than variation observed between domestication groups. The values observed in this study were higher than those previously reported^16,17^, probably because of the larger genotypic variance existing in our panel, as well as the homogeneous conditions over the experiments. Seven bacterial phylotypes displayed strong and significant broad sense heritability after correcting for multiple testing (FDR procedure, q < 0.05). Those phylotypes belong to the Actinobacteria (two of them), the Gemmatimonadetes^1^, the Nitrospirae^1^, the Proteobacteria^2^ and the Verrucomicrobia^1^ phyla. Among those, members of the Rhizobiales order exhibited the highest heritability estimates with 28.2% of their relative abundance explained by differences in host genotype. Interestingly, Rhizobiales are well-known beneficial partners in plant–microbe interactions^58^. Members of the Nitrospiraceae family also showed high heritability estimates (24.8%). Of note, there are several studies describing the role of Nitrospiraceae in nitrogen cycling but, to our knowledge, none has described this taxa as significantly impacted by plant roots. Finally, the Gemm 1 clone within the Gemmatimonadetes phylum is of particular interest because it showed both variations between domestication groups but also significant genotypic variation within each group with a heritability estimate of 23.2%. Unfortunately, despite being found in a variety of soils, little is known about its ecology.

Several fungal phylotypes also displayed significant variation related to the identity of the host genotype. Probably the most interesting one is the Ophiostomataceae phylotype containing the *Ophiostoma* and *Raffaela* genera, which were also impacted by domestication. The relative abundance of Ophiostomataceae family members was strongly dependent on the host genotype (26.6%), especially within the *dicoccoides* subspecies. In this subspecies, the counts variation attributed to this phylotype ranged between ~ 50 for genotype 46309 to ~ 1,200 for genotype 46499 while it showed a much smaller range of variation in the three other domestication groups. The abundance of the total rhizosphere associated microbial community, as well as those of the N-cycle microbial guilds were not significantly influenced by the host genotypic variation.

### Relationships between plant phenotypes and microbial communities

**Figure 4:**
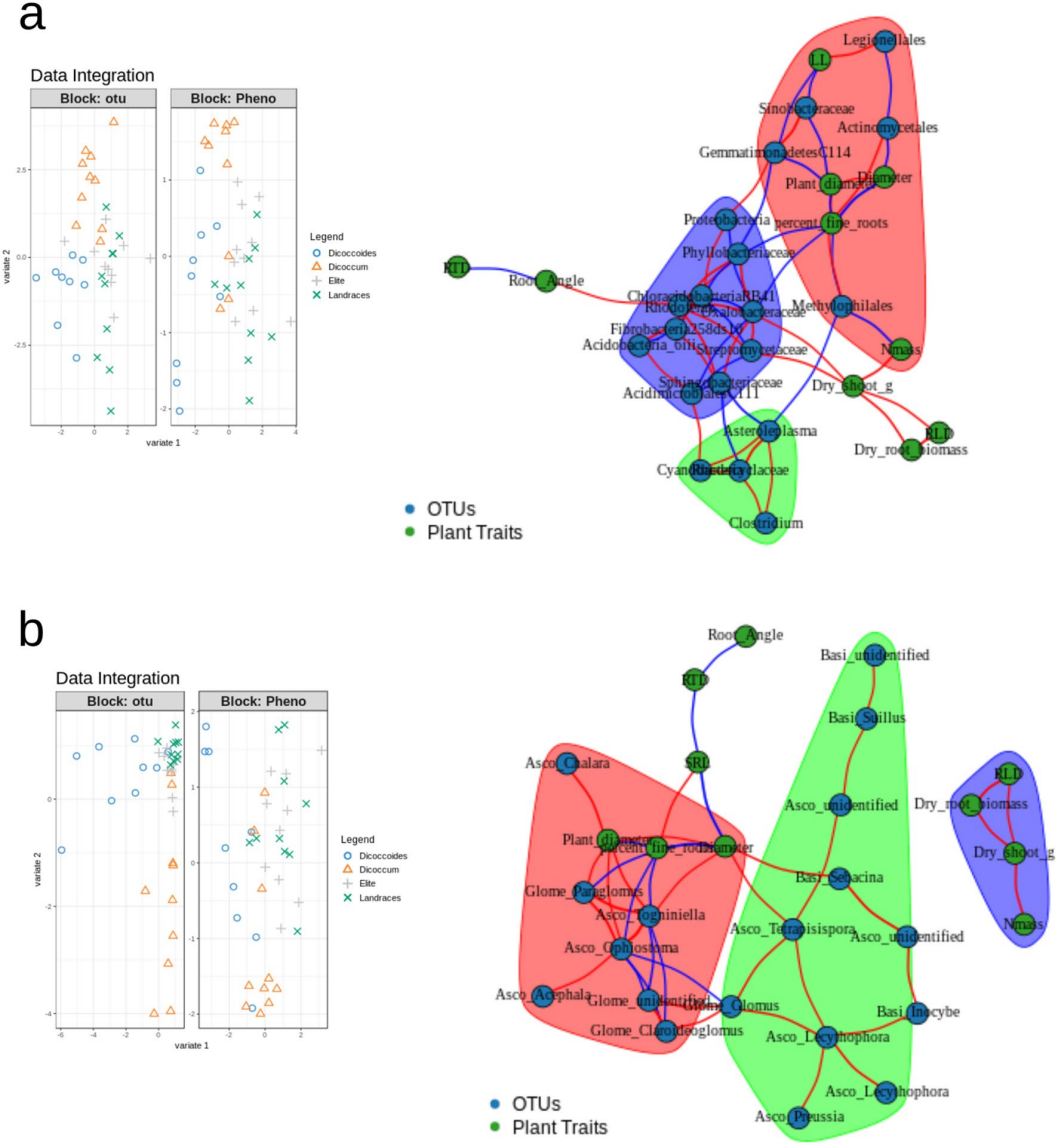
Network visualization of associations between bacterial and fungal OTUs abundances, and ten above and belowground plant traits across the four domestication groups. Green and blue nodes correspond to plant phenotypic traits and bacterial (**a**) or fungal (**b**) OTUs, respectively. Edges indicate positive (red) or negative correlations (blue) as defined by Pearson’s correlation coefficients r > 0.4 or r < − 0.4, respectively. Different modules are represented by different colors (green, red, and blue). Left panels are showing discrimination of the samples based only on the sub-selected OTUs and plant traits that correlate.

We sought to determine whether the variations in the rhizosphere microbiome were related to changes in wheat phenotypic traits during domestication. The phenotypic distance between plants was positively correlated to differences in composition of rhizosphere bacterial communities (Mantel R test, R = 0.25, p = 0.02), but not to the overall fungal community composition (Mantel R test, R = 0.12, p = 0.14). We then combined the microbial community composition data with the plant phenotypic trait dataset in order to detect covariation in those datasets that might be related to domestication using a DIABLO approach. When looking at both microbial communities, a correlation between the most discriminant bacterial and fungal OTUs, and plant phenotypic traits was observed on the 1st component. This 1st component was mainly discriminating *T. t. spp. dicoccoides* accessions from the others while the 2nd one discriminated most of the *T. t. spp. dicoccum* accessions from the others (Fig. 4). We identified nine bacterial and six fungal OTUs that correlated with seven plant phenotypic traits (Table 1). Figure 4 highlights links between those OTUs and plant phenotypic traits indicating negative and positive correlations (|r|> 0.4, assessed via DIABLO similarity scores that are analogous to Pearson’s correlation coefficients). Plant diameter was the trait exhibiting the strongest associations with microbial taxa, being both positively correlated with the relative abundance of unknown members the Basidiomycota phylum (r = 0.61), and negatively correlated with the relative abundance of members C114 of the Gemmatimonadetes phylum (r = − 0.57). Previous study suggested that differences in root architecture can also determine how effective Glomeraceae are at reducing fungal pathogen abundance^59^. This is consistent with our findings showing that potential plant pathogen fungi were both negatively correlated to the percentage of fine roots, namely here Togniniella and Ophiostoma, and to the proportion of beneficial Glomeraceae, namely here Clareidoglomus, Glomus and an unidentified member of the Glomeromycota (Fig. 4). Taken together, our results showed that the abundances of several fungal taxa impacted by domestication were also negatively related to the percentage of fine roots, which indicate that changes in plant morphology, especially in the root architecture during domestication, might have driven changes in the composition of the rhizosphere microbiota.

**Table 1:** Correlations between plant traits and the relative abundances of microbes related to domestication.

Similarity score^a^	Plant diameter	Diameter	Leaf longevity	Root angle	N mass	Percent fine roots	Dry shoot
Actinobacteria:Actinomycetales	–	− 0.54	–	–	–	0.49	–
Actinobacteria:Streptomycetaceae	–	–	–	–	–	–	0.49
Ascomycota:Ophiostoma	0.42	–	–	–	–	− 0.42	–
Ascomycota:Tetrapisispora	–	0.42	–	–	–	–	–
Ascomycota:Togniniella	0.53	0.40	–	–	–	− 0.52	–
Basidiomycota:Sebacina	–	0.53	–	–	–	–	–
Basidiomycota:unidentified	0.61	–	–	–	–	–	–
Gemmatimonadetes:C114	− 0.57	–	− 0.43	–	–	–	–
Glomeromycota:Paraglomus	0.47	–	–	–	–	− 0.49	–
Proteobacteria:Legionellales	–	–	0.53	–	–	–	–
Proteobacteria:Methylophilales	0.41	–	–	–	− 0.55	− 0.41	–
Proteobacteria:Oxalobacteraceae	–	–	–	–	–	− 0.40	0.44
Proteobacteria:Phyllobacteriaceae	–	–	–	–	–	− 0.47	–
Proteobacteria:Rhodoferax	–	–	–	0.41	–	–	–
Proteobacteria:Sinobacteraceae	− 0.52	–	− 0.47	–	–	–	–

^a^Note that similarity scores in DIABLO are analogous to Pearson’s correlation coefficients.

## Conclusion

Altogether, our findings indicate that domestication has shaped the rhizosphere associated microbiota in wheat. The impact of domestication was stronger on fungi with a large decrease of potential pathogens associated with an increase of obligate plant symbionts belonging to the glomeromycota phylum, that were related to a change in the morphology of the root system between wild and cultivated emmer. The depletion of microbes potentially important for plant nutrient availability, e.g. nitrifying bacteria as well as arbuscular mycorrhizal fungi, between primitive domesticated forms and modern accessions suggests that the Green revolution, which relied on chemical fertilizers, might have caused a decrease in the association between tetraploid wheat and its rhizosphere microbiota. Nevertheless, the strong genotypic variation that we observed within each domestication group, indicates that there might be room for genetic improvement via introgression of ancestral plant rhizosphere-beneficial microbe associations.

## .

## Supplementary information

Supplementary Information 1.
