# Supplementary material for: Domestication-driven changes in plant traits associated with changes in the assembly of the rhizosphere microbiota in tetraploid wheat
Source: Sci Rep. 2020 Jul 22;10:12234. doi: 10.1038/s41598-020-69175-9 (PMC7376052; doi:10.1038/s41598-020-69175-9)

Supplementary Information for

# Domestication-driven changes in plant traits associated with changes in the assembly of the rhizosphere microbiota in tetraploid wheat

***Authors:*** Aymé Spor[1*#], Agathe Roucou[2,3*], Arnaud Mounier[4], David Bru[1], Marie-Christine Breuil[1], Florian Fort[5], Denis Vile[2], Pierre Roumet[6], Laurent Philippot[1#] and Cyrille Violle[2]

[#]**Corresponding authors:** A. Spor: ayme.spor@inrae.fr & L. Philippot: laurent.philippot@inrae.fr

**This PDF file includes:**

Figures S1 to S3
Tables S1

**Supplementary Figure legends**

**Fig. S1.** Impact of the rhizosphere environment on bacterial and fungal communities composition. Constrained analyses of principal coordiantes of bacterial (a) and fungal (b) communities. Black and red dots correspond to bulk and rhizosphere samples, respectively. (c) mean (and s.e) OTU counts of bacterial and fungal classes enriched in the rhizosphere (blue bars) compared to bulk soil (red bars) (ANOVA, $p < 0.05$). (d) mean (and s.e) OTU counts of bacterial and fungal classes depleted in the rhizosphere (blue bars) compared to bulk soil (red bars) (ANOVA, $p < 0.05$).

**Fig. S2.** Impact of domestication on fungal communities composition. (a) Fungal OTUs displaying the type-1 (a) and the type-2 (b) patterns. Black, blue, white and red dots correspond to *dicoccoides*, *dicoccum*, *durum* elite varieties and *durum* landraces, respectively. Mean and standard errors are represented.

**Fig. S3.** (a) Bacterial cooccurence network across the bulk soil samples. Edges indicate positive (green) or negative correlations (red) as defined by Spearmann's correlation $|\rho| > 0.6$. Node colors differ according to OTUs phylum assignment. (b) Venn diagram illustrating the number of nodes shared across the four domestication groups and the bulk soil bacterial cooccurence networks.

**Table S1. List of the 40 tetraploid wheat accessions used in this study.**

| Sub-species | N | Collection |
|---|---|---|
| *T. turgidum* ssp. *dicoccoides* | 46499 | ICARDA |
| (ancestral form) | 46491 | ICARDA |
| | 46323 | ICARDA |
| | 46309 | ICARDA |
| | *46310* | *ICARDA* |
| | 46501 | ICARDA |
| | 46453 | ICARDA |
| | 46253 | ICARDA |
| | 467014 | USDA |
| | 352324 | USDA |
| *T. turgidum* ssp. *dicoccum* | | |
| (first domesticated form) | 45383 | ICARDA |
| | 45351 | ICARDA |
| | 45239 | ICARDA |
| | 45354 | ICARDA |
| | 45280 | ICARDA |
| | 45441 | ICARDA |
| | 352365 | USDA |
| | 319868 | USDA |
| | cwi17084 | CIMMYT |
| | 415152 | USDA |
| *T. turgidum* ssp. *durum* | | |
| (Landrace) | | |
| (durum wheat before Green Revoluton) | 495 | INRA Montpellier |
| | 84866 | ICARDA |
| | 97512 | ICARDA |
| | 95920 | ICARDA |
| | 82697 | ICARDA |
| | 82702 | ICARDA |
| | 82768 | ICARDA |
| | 82715 | ICARDA |
| | 82726 | ICARDA |
| | (maria)B6R | 580 |
| *T. turgidum* ssp. *durum* (Elite) | | |
| (durum wheat after Green Revolution) | AGATHE | GEVES |
| | BRUMAIRE | GEVES |
| | DURGAMM | GEVES |
| | NITA | GEVES |
| | ROMEO | GEVES |
| | PRIMADUR | GEVES |
| | ARBOIS | GEVES |

| | |
|---|---|
| NEODUR | GEVES |
| IXOS | GEVES |
| DURENTAL | GEVES |

ICARDA (International Center for Agricultural Research in the Dry Areas) Beirut, Lebanon.
USDA-ARS (US Department of Agriculture) National Small Grains, wheat collection, USA.
CIMMYT (International Maize and Wheat Improvement Center), Mexico
GEVES (French Group for the Study and Control of Varieties and Seeds), France
INRA (French National Institute of Agricultural Research) wheat collection, Montpellier

Supp figure 1.

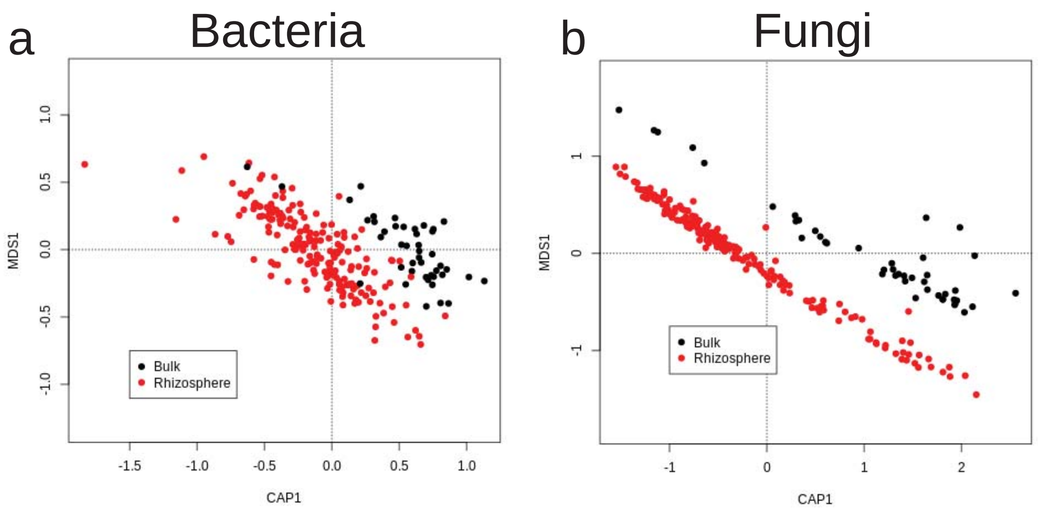

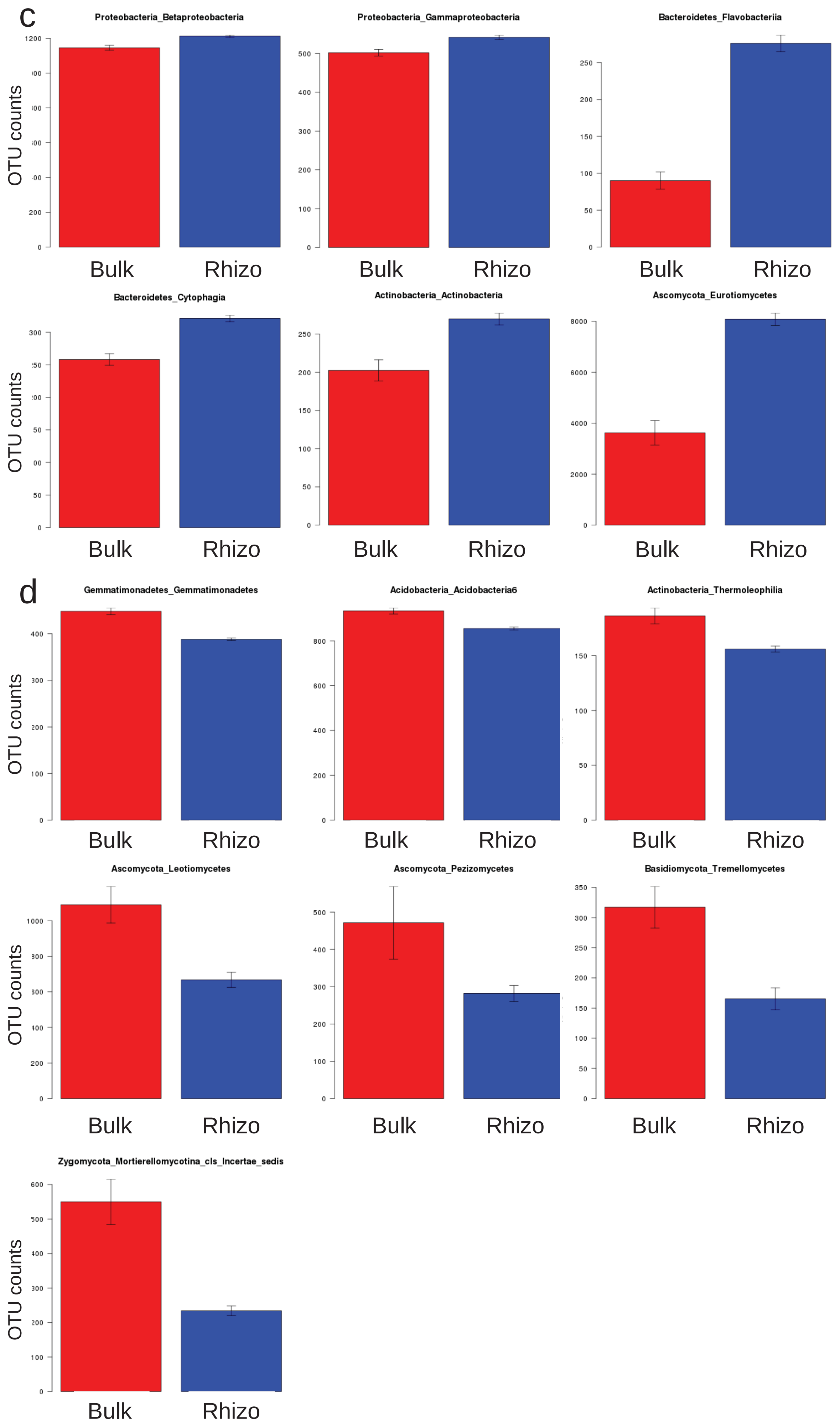
c
Proteobacteria_Betaproteobacteria
Proteobacteria_Gammaproteobacteria
Bacteroidetes_Flavobacteriia
Bacteroidetes_Cytophagia
Actinobacteria_Actinobacteria
Ascomycota_Eurotiomycetes
d
Gemmatimonadetes_Gemmatimonadetes
Acidobacteria_Acidobacteria6
Actinobacteria_Thermoleophilia
Ascomycota_Leotiomycetes
Ascomycota_Pezizomycetes
Basidiomycota_Tremellomycetes
Zygomycota_Mortierellomycotina_cls_Incertae_sedis
OTU counts
Bulk
Rhizo

# Supp figure 2.

a

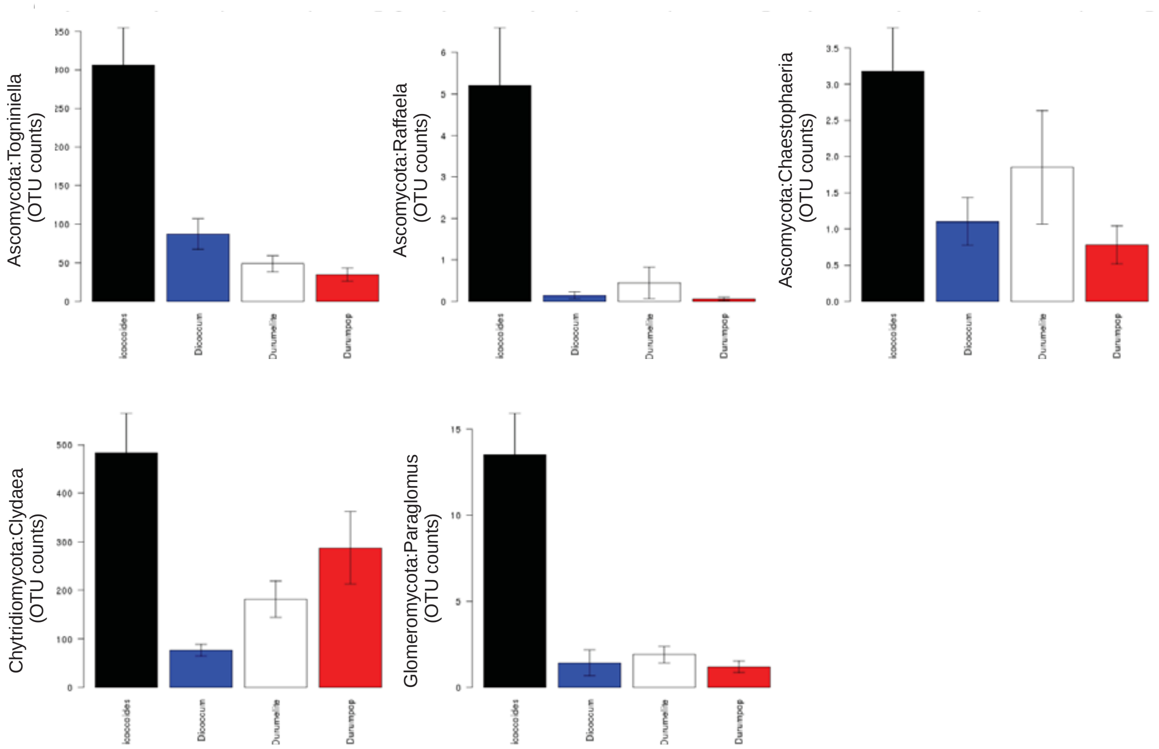


b

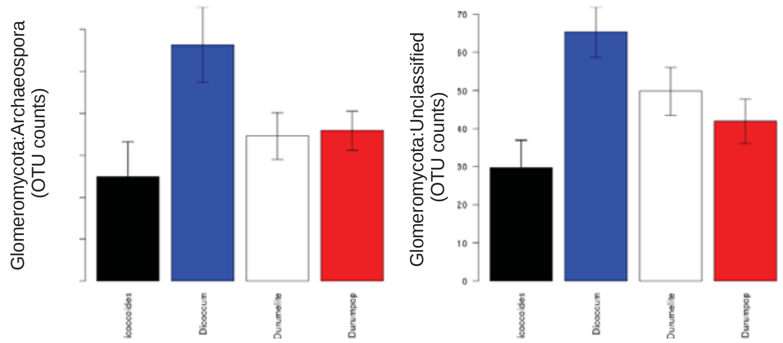

# Supp figure 3.

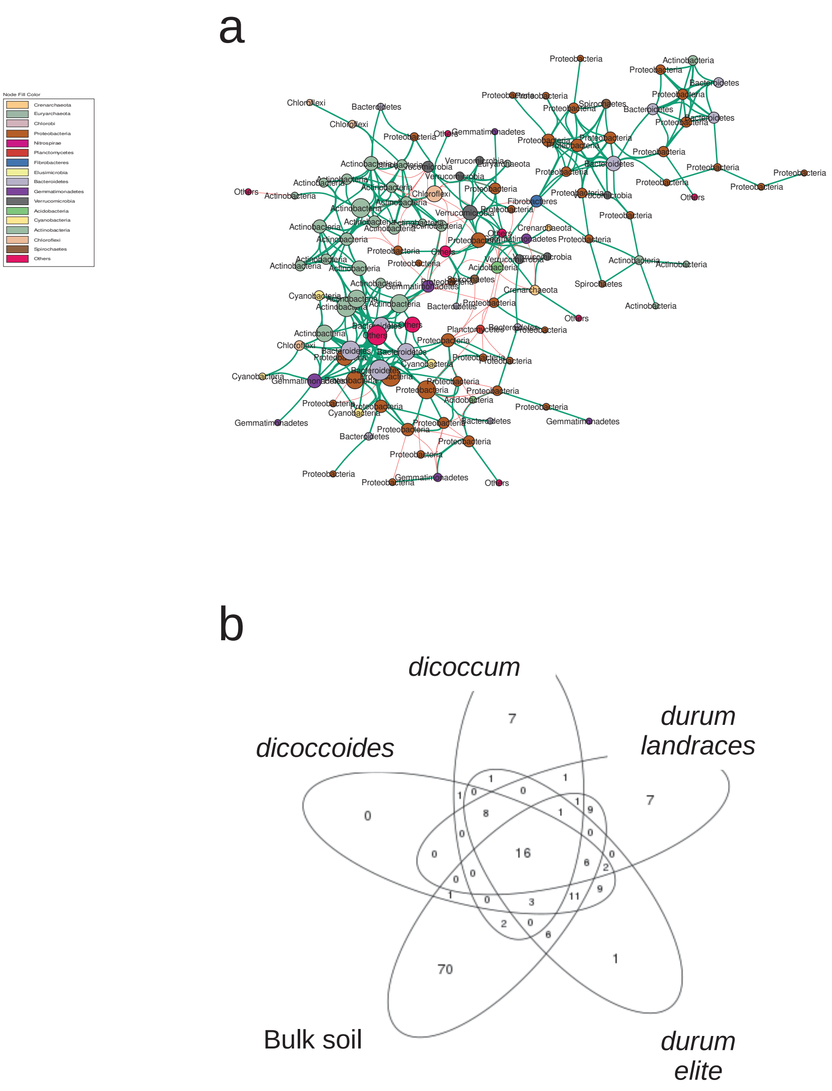

Supplement: Supplementary file 1 — Supplementary Information 1. [file 41598_2020_69175_MOESM1_ESM.pdf]
